# Open-mindedness can decrease persuasion amongst adolescents: The role of self-affirmation

**DOI:** 10.1111/bjhp.12090

**Published:** 2014-01-29

**Authors:** Anna Good, Peter R Harris, Donna Jessop, Charles Abraham

**Affiliations:** 1 School of Psychology, University of SussexFalmer, UK; 2 University of Exeter Medical SchoolExeter, UK

**Keywords:** self-affirmation, defensiveness, health-risk information, physical activity

## Abstract

**Objectives:**

Self-affirmation (e.g., by reflecting on important personal values) has been found to promote more open-minded appraisal of threatening health messages in at-risk adults. However, it is unclear how self-affirmation affects adolescents and whether it has differential effects on the impact of these messages amongst those at relatively lower and higher risk. The current study explored moderation by risk.

**Design:**

Participants were randomly assigned to either a self-affirmation or a control condition before receiving a health message concerning physical activity.

**Methods:**

Older adolescents (*N* = 125) completed a self-affirmation or control writing task before reading about the health consequences of not meeting recommendations to be physically active for at least 60 min daily. Most of the sample did not achieve these levels of activity (98%, *N* = 123). Consequently, the message informed these participants that – unless they changed their behaviour – they would be at higher risk of heart disease. Participants completed measures of responses to the message and behaviour-specific cognitions (e.g., self-efficacy) for meeting the recommendations.

**Results:**

For relatively inactive participants, self-affirmation was associated with increased persuasion. However, for those who were moderately active (but not meeting recommendations), those in the self-affirmation condition were less persuaded by the message.

**Conclusions:**

Whilst self-affirmation can increase message acceptance, there are circumstances when the open-mindedness it induces may decrease persuasion. The evidence provided in this study suggests that caution may be needed when recommendations are challenging and it could be considered reasonable to be sceptical about the need to change behaviour.

**Statement of contribution**

**What is already known on this subject?**

- Self-affirmation can facilitate open-mindedness and sensitivity to whether health messages suggest high or low risk on the basis of current behaviour.

**What does this study add?**

- Demonstrates that self-affirmation effects can be moderated by the extent of failure to meet recommendations.
- Shows that self-affirmation can be associated with less persuasion when challenging health guidelines are used.

## Background

Messages that imply personal inadequacy (e.g., failure to take enough exercise) are often rejected or resisted, especially amongst those most at risk (Good & Abraham, 2007). According to self-affirmation theory (Steele, 1988), these responses are elicited because our overarching desire for positive self-perception is threatened when we are told we are not acting sensibly or taking care of ourselves (cf. Harris & Epton, 2009). So how can such resistance be overcome? Steele's theory suggests that defensiveness is eliminated or at least reduced when the self is affirmed, for example by completing a relevant values scale or writing a brief essay on an important value. Consistent with this, reviews have concluded that affirming important values or personal attributes can facilitate more dispassionate appraisals of threatening health messages and other unwelcome information (Aronson, Cohen, & Nail, 1999; Harris & Epton, 2009; McQueen & Klein, 2006; Sherman & Cohen, 2006). Such appraisals have generally resulted in greater message acceptance, because researchers have aimed to present ‘good or high-quality information that should be accepted after a truly objective analysis’ (Munro & Stansbury, 2009, p. 1144).

In practice, however, health guidance may be focussed on what is ideal, and little consideration may be given to whether or not it is likely to be perceived as reasonable and acceptable to its recipients. For example, at least 80% of the UK population fails to achieve the ‘five a day’ recommendation for fruit and vegetable intake (Henderson, Gregory, & Swan, 2002; Jackson *et al*., 2005), and it is considered by many to be unrealistic (Anderson & Cox, 2000; Daborn, Dibsall, & Lambert, 2005). Likewise, widely publicized safe alcohol consumption guidelines of no more than 3–4 units for men and 2–3 units for women have been met with incredulity (Heather, 2009) and are not considered helpful by those wishing to regulate their alcohol intake (de Visser & Birch, 2012). In a review, Heather noted that ‘in all probability, people's experience of everyday life will lead them intuitively to regard such warnings as non-sense … and dismiss public health messages about drinking entirely’ (pp. 226–227). In examples such as these, message rejection may be understandable and is arguably rational.

Evidence indicates that self-affirmation promotes open-mindedness and, thereby, facilitates attention to the merits and demerits of presented messages. Correll, Spencer, and Zanna (2004) found that relative to those in a no-affirmation control condition, self-affirmed participants were more attuned to the strength of counter-attitudinal arguments. In a health context, this can have important consequences. For example, Klein, Harris, Ferrer, and Zajac (2011) report evidence suggesting that the quality of message arguments determines whether self-affirmation is associated with increased or decreased feelings of vulnerability and intentions to change. When participants were presented with a message citing less reliable evidence supporting a link between caffeine consumption and fibrocystic breast disease, those who were self-affirmed felt less susceptible to the disease and expressed lower intentions to reduce their caffeine intake relative to those in the control condition. Similarly, when Zhao and Nan (2010) used a weak message about the benefits of stopping smoking, self-affirmation produced greater negativity in judgements of this message.

Self-affirmation should also enhance judgement of whether or not it is appropriate to feel worried or reassured by a health message. Griffin and Harris (2011) found that self-affirmation sensitized or ‘calibrated’ participants’ responses to the personal relevance of a message about safe consumption levels for tuna. Concern was reduced amongst those eating within guidelines, but increased amongst those made aware that they had exceeded them. Similarly, using a message linking breast cancer to alcohol intake, Harris and Napper (2005) demonstrated that self-affirmation strengthened links between whether or not stated limits were exceeded and perceived susceptibility. Initial findings suggest that these effects may extend to behaviour, with self-affirmation leading to greater sensitivity to personal risk when deciding whether to take an online test for susceptibility to diabetes (van Koningsbruggen & Das, 2009). van Koningsbruggen and Das interpret such findings as indicating that self-affirmation may have ‘adverse effects’, but, as Harris and Epton (2009) note, self-affirmation ‘is not a technique for increasing persuasion. Instead, self-affirming affords more objective appraisal of existing information allowing it to ‘speak for itself’ … whether that results in message acceptance depends on the quality of the information’ (p. 441).

Although there is evidence that self-affirmation may calibrate, or moderate, responses to threat messages according to whether individuals are informed that they are at low or high risk, no research has examined whether self-affirmation effects vary as a function of how reasonable it is to suggest that they need to change behaviour. Guidelines that result in quite healthy individuals being told that they are at risk may be especially prone to rejection following self-affirmation. Consider, for example, the advice of the National Institute for Health and Care Excellence that there should be a long-term campaign encouraging 5- to 18-year-olds to engage in moderate-to-vigorous physical activity (MVPA) for at least 60 min each day (Mayor, 2009). The vast majority of older adolescents fail to meet these recommendations – but there is wide variation in the extent of this ‘failure’. Many within this age group do little or no MVPA, whereas others meet or exceed the 150 min per week that are recommended for adults (Woods, Tannehill, & Walsh, 2010). It seems appropriate to suggest that ‘low exercisers’ are insufficiently active, but those who are active, but yet fail to meet these stringent guidelines, might reasonably reject the argument that it is necessary or even feasible to increase their physical activity levels to at least 60 min a day. Indeed, whilst physical inactivity has very high population attributable risks (e.g., for diabetes, colon cancer, and cardiovascular disease; Powell & Blair, 1994), with adolescence being a critical period for preventing age-related decreases in activity and establishing habits for the future (Janz, Burns, & Levy, 2005; Riddoch *et al*., 2004; Twisk, 2001), the evidence for a particular threshold concerning the amount of physical activity is debatable (cf. Twisk, 2001).

As self-affirmation appears to increase sensitivity to information validity and personal relevance, the degree to which individuals fall short of recommendations has the potential to moderate its effects. When guidelines are provided, open-minded acceptance of the ease and necessity of behaviour change can occur in reference to the overall behaviour (e.g., increasing physical activity) or specified behavioural targets (e.g., getting at least 60 min of physical activity each day). It is probable that those who are relatively inactive will focus on being *more* physically active, whereas those closer to achieving the recommendations might be more focused on exactly *how much* activity is recommended to avert the threat – particularly if they are in an accuracy-oriented mindset induced by self-affirmation. On this basis, self-affirmation can reasonably be expected to exert the typical effect of increasing message acceptance amongst those who are less active, but to facilitate open-minded rejection amongst those who are already relatively active.

### The current study

This study is the first to test whether the gap between actual and recommended behaviour moderates the effects of self-affirmation. In particular, the study focused on understanding how self-affirmation influences responses of those whose behaviour could reasonably be considered healthy, but who are informed that their failure to meet recommendations could increase their susceptibility to health risks. These issues are explored in the context of providing older adolescents (16- to 18-year-olds) with Department of Health (2011) physical activity guidelines for their age group. In line with typical health promotion messages, participants were presented with information emphasizing that it is easy to meet recommendations and that whether or not this is achieved influences the risk of future ill-health.

We predict that the extent to which physical activity falls short of recommended levels will moderate the effect of self-affirmation on message derogation and acceptance, perceived risk, response- and self-efficacy, behavioural expectations, and self-reported physical activity. We hypothesize that amongst less active participants, self-affirmation will be associated with more positive responses in terms of (1) perception of the message (i.e., less message derogation, more acceptance), (2) behaviour-specific cognitions (i.e., higher perceived risk, response-efficacy, self-efficacy, and expectations for meeting the recommendations), and (3) higher levels of self-reported activity 1 week later. Conversely, we hypothesize that, amongst more active participants, self-affirmation will encourage rejection of the suggestion that increasing physical activity to least 60 min each day is necessary or feasible, producing the opposite pattern of findings on these measures.

## Method

### Participants

One hundred and twenty-five adolescents were recruited from sixth form colleges in Sussex, United Kingdom. Two participants (2%) were excluded from statistical analysis as they met the physical activity recommendations of at least 60 min of physical activity 7 days/week. The remaining participants (*N* = 123) were 43 boys and 80 girls aged 16–18 years (*M* = 16.57, *SD* = 0.60), the majority of whom were British (91%). Eighty-five participants (69%) completed time 2 (T2) measures. There were no significant differences between those who did and did not complete the T2 measures in terms of the condition they were assigned to (*p* = .95, Cramér's *V* = .01) or time 1 (T1) self-reported physical activity (*p* = .09, η^2^ = .02)^1^; however, those who completed measures at both time points were slightly younger (*M* = 16.48, *SD* = 0.57) than those who did not complete the follow-up (*M* = 16.79, *SD* = 0.62), *F*(1, 121) = 7.22, *p* = .01, η^2^ = .06, and comprised a larger proportion of females, χ^2^(1, *N* = 123) = .89, *p* = .02, Cramér's *V* = .21.

### Procedure and design

**Figure 1:**
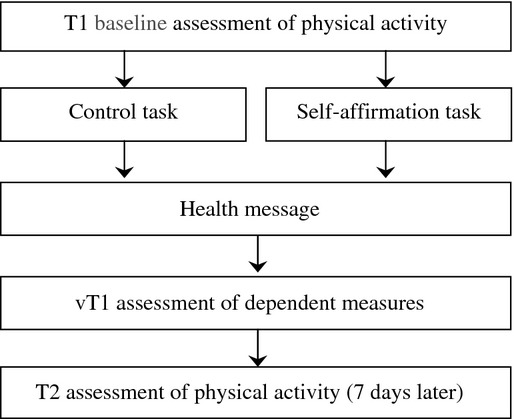
Overview of study design and procedure.

This prospective randomized study comprised an online questionnaire and a brief follow-up 1 week later. Using an automated algorithm within the website programming, participants were randomly assigned to either the control (*n* = 62) or self-affirmation condition (*n* = 61) in a between-subjects design. All participants were then exposed to information about physical activity and heart disease before completing T1 measures. One week later, participants were invited by email to report their physical activity over the intervening 7 days. An overview of the study design and timings can be seen in Figure1.

### Materials

After completing initial demographic items, participants were asked about the number of days they had engaged in moderate-to-vigorous physical activity for at least 60 min ‘in the past 7 days’, and ‘over a typical or usual 7-day period’, using definitions from the Department of Health (DoH, 2011). These items were adapted from the PACE+ Adolescent Physical Activity Measure (Prochaska, Sallis, & Long, 2001), which has good reliability and convergent validity with objective physical activity data (Prochaska *et al*., 2001). In the current study, the interitem correlation was .75.

### Self-affirmation manipulation

Using a standard self-affirmation procedure (based on Cohen, Aronson, & Steele, 2000), participants then wrote about the personal importance of their most important value (SA condition) or about why their least important value might be of importance to someone else (control condition). To check that participants rated the value they wrote about as more important in the self-affirmation than the control condition, they were asked*:* ‘How important to you is the value that you selected to write about?’ (7-point scale: *Extremely unimportant* – *Extremely important*).

### Health message

Following the manipulation, participants were informed of the recommendations for their age group (at least 60 min of physical activity over the course of each day, which should be a mix of moderate and vigorous physical activity). On the next page, there was an information menu containing four links. Two links related to why young people should get the recommended activity. These ‘why links’ emphasized threat and response-efficacy (‘the risk of heart disease’; ‘reducing the risk of heart disease’). Two links emphasized self-efficacy and problem-solving for meeting recommendations (‘how you can get the recommended amount of physical activity through different activities’; ‘how to overcome barriers to getting the recommended amount of physical activity’). The presentation of the links within the table was counterbalanced so that the two ‘why information’ links were on the left and the two ‘how information’ links on the right, or *vice versa*. All participants opened the four links.

### Response to the message and behaviour-specific cognitions

Responses to the presented information were assessed using *message derogation* and *acceptance* items adapted from Ruiter, Verplanken, Kok, and Werrij (2003): (*derogation*-‘What did you think about the information about physical activity that you just read?’ ‘It was exaggerated’, ‘It tried to manipulate my feelings’, interitem correlation = .55; *acceptance*-‘It was persuasive’; 1 = *totally disagree*, 7 = *totally agree*).

Four measures of cognitions specifically relating to the recommended behaviour change were assessed: *perceived risk* was measured using a single item (‘My chances of developing heart disease in the future are’: 1 = *not at all strong*, 7 = *very strong*). Three- and four-item scales anchored at 1 = *strongly disagree* and 7 = *strongly agree* were used to measure *response-efficacy* (‘Getting at least 60 min of moderate-vigorous physical activity a day is effective in reducing the risk of heart disease/works in preventing heart disease’, ‘If I were to get at least 60 min of moderate-vigorous physical activity a day I would lessen my chances of developing heart disease’. Average inter-item correlation = .56) and *self-efficacy* (‘I feel confident in my ability to get at least 60 min of moderate-vigorous physical activity a day over the next 7 days’, ‘I am discouraged from getting at least 60 min of moderate-vigorous physical activity a day over the next 7 days because I feel unable to do so’, ‘I feel confident in my ability to get at least 60 min of moderate-vigorous physical activity a day over the next 7 days’, ‘Getting at least 60 min of moderate-vigorous physical activity a day over the next 7 days would be easy for me’. Average interitem correlation = .55; adapted from Milne, Orbell, and Sheeran, 2002). *Behavioural expectations* were then reported (‘On how many of the next 7 days do you expect you will engage in moderate-vigorous physical activity for at least 60 min per day?’: 0–7 days).

### Physical activity at T2

One week after the T1 questionnaire, participants were again asked to report the number of days in the previous 7 days in which they had engaged in MVPA for at least 60 min (0–7).

### Data analyses

Multiple imputation using all variables was applied using IBM SPSS Statistics for Windows (Version 20.0; IBM Corp., Armonk, NY, USA) to deal with data missing (due to attrition) on the T2 self-reported behaviour item. This technique generates multiple values for respective points of missing data to account for missing-data uncertainty, creating multiple data sets, and introducing between-imputation variance. For the present study, five imputed data sets were created (Graham, Olchowski, & Gilreath, 2007).

Data were analysed using moderated hierarchical regression analysis (Aiken & West, 1991). To control for the order in which ‘how’ and ‘why’ links were presented, this variable was entered in step 1, along with SA condition (1 = *self-affirmation*, 0 = *control*) and physical activity.^2^ In step 2, interactions between condition and physical activity were examined by entering the product of these first-order variables. Rather than arbitrarily defining ‘low’, ‘moderate’, and ‘high’ activity values, significant interactions were probed using the Johnson–Neyman technique (Johnson & Neyman, 1936; see Bauer & Curran, 2005; Hayes & Matthes, 2009). This technique enables identification of the particular levels of the moderator at which there are significant differences according to condition.

## Results

The average number of days on which participants reported getting at least 60 min of physical activity at T1 was substantially below the recommended 7 days a week (*M* = 2.19, *SD* = 1.54), with more than half (55%) meeting the daily recommendation on 2 days a week or fewer. There was a slight increase overall in self-reports of physical activity from baseline to T2 (*M* = 2.53, *SD* = 1.88), *F*(1, 699) = 71.58, *p* < .001, η^2^ = .01. Randomization was successful, with no difference in the physical activity levels (*p* = .81), age (*p* = .41), or gender of participants (*p* = .62) in the two conditions. Furthermore, as predicted, those in the SA condition rated the value they wrote about as being significantly more important (*M* = 5.05) than those in the control condition (*M* = 3.08), *F*(1, 120) = 26.14, *p* < .001, 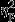 = .18. Means and standard deviations for all measures are shown by condition in Table 1.

**Table 1:** Means (and *SD*s) for all measures

	Control (*N* = 62)	Self-affirmation (*N* = 61)
T1		
Message acceptance	4.52 (1.52)	4.30 (1.62)
Message derogation	3.69 (1.37)	3.70 (1.50)
Perceived risk	3.52 (1.26)	3.43 (1.20)
Response-efficacy	5.33 (0.97)	5.14 (1.09)
Self-efficacy	4.39 (1.48)	4.59 (1.16)
Behavioural expectations	3.03 (1.58)	2.74 (1.66)
Physical activity scale	2.15 (1.40)	2.22 (1.67)
Physical activity (past 7 days)	2.06 (1.35)	2.26 (1.72)
T2		
Physical activity (past 7 days)	2.56 (1.75)	2.54 (2.03)

### Physical activity as a moderator of self-affirmation effects

**Figure 2:**
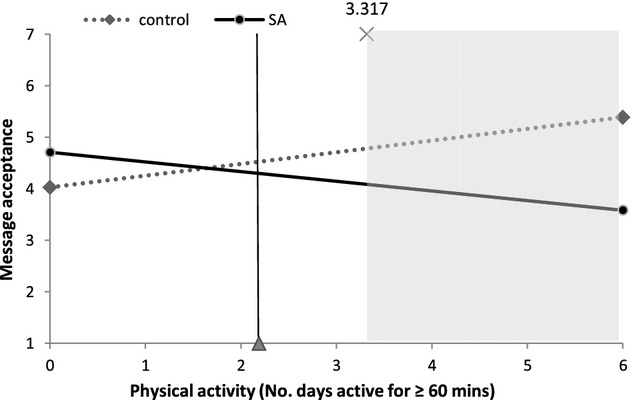
The effect of self-affirmation on message acceptance for participants at different levels of physical activity.

**Figure 3:**
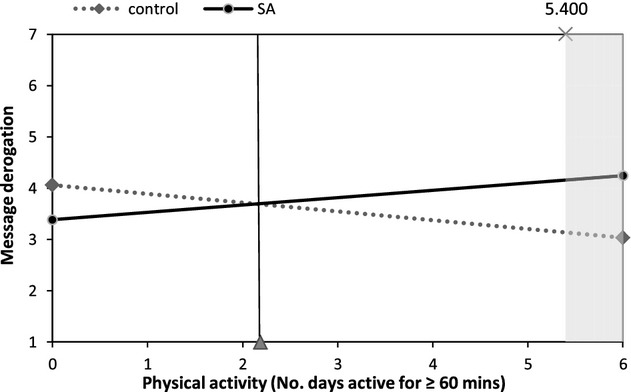
The effect of self-affirmation on message derogation for participants at different levels of physical activity.

**Figure 4:**
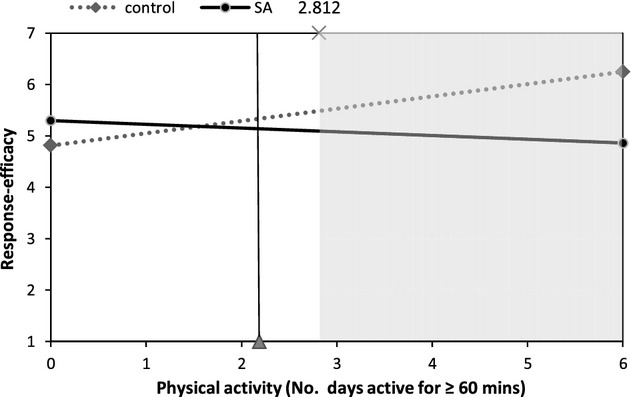
The effect of self-affirmation on response-efficacy for participants at different levels of physical activity.

**Figure 5:**
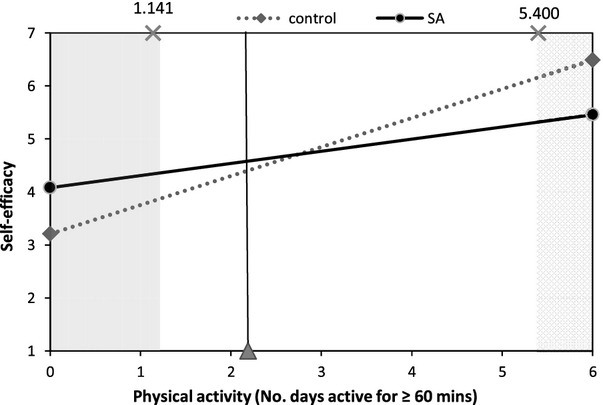
The effect of self-affirmation on self-efficacy for participants at different levels of physical activity.

Figures 2–5 illustrate interactions between self-affirmation and physical activity. The shaded area on each graph highlights the region of significance, and the vertical line indicates the mean level of activity (at least 60 min of activity on 2.2 days/week). As predicted, the extent to which participants fell short of recommendations moderated the effect of self-affirmation on responses to message and behaviour-specific cognitions. It is noteworthy that, with the exception of behavioural expectations, there were no significant main effects of self-affirmation on outcomes. For behavioural expectations, participants in the control condition reported higher behavioural expectations than did those in the self-affirmation conditions (*M*s 3.03 and 2.74, respectively), *b* = −.35, *t*(119) = −2.04, *p* = .04.

The regression analysis on *message acceptance* revealed a significant interaction between self-affirmation and physical activity, *b* = −.42, *t*(118) = −2.24, *p* = .03. As can be seen in Figure2, a regions-of-significance test showed that amongst active participants (60 min or more of MVPA on at least 3.3 days/week), self-affirmation was associated with less acceptance of the message.

A similar pattern of results was found for *message derogation*, with a marginally significant self-affirmation × activity interaction, *b* = .32, *t*(118) = 1.83 *p* = .07. Although there were no regions of significance at the *p* ≤ .05 level, the association between condition and message derogation approached significance (*p* ≤ .10) for active participants (60 min or more of MVPA on at least 5.4 days a week).

The regression analysis for response-efficacy also revealed an interaction between self-affirmation and activity, *b* = −.32, *t*(118) = −2.57, *p* = .01. As predicted, amongst more active participants, self-affirmed participants had lower response-efficacy than those in the control condition for those getting 60 min or more of MVPA on at least 2.8 days/week.

Ratings of self-efficacy followed a similar pattern. Examination of the significant self-affirmation × activity interaction, *b* = −.32, *t*(118) = −2.26, *p* = .03, revealed regions approaching significance at both low and high levels of activity. In line with findings for other measures, self-affirmation was associated with lower self-efficacy for highly active participants (60 min or more of MVPA on at least 5.4 days; *p* ≤ .10) but, as predicted, higher self-efficacy for those who were less active (1.1 days a week or less; *p* ≤ .05).

In summary, there was good support for the prediction that self-affirmation effects on responses to the message would be moderated by baseline levels of physical activity. Within measures of behaviour-specific cognition, this moderating effect was found for response- and self-efficacy, but not for perceived risk (*p* = .50) or behavioural expectations (*p* = .16). There was no significant interaction between physical activity and self-affirmation for physical activity at T2 (*p* = .95).

## Discussion

Responses to health warnings can be inappropriate because of under-reaction due to defensiveness or overreaction due to oversensitivity to emotional arousal. Previous research has shown that self-affirmation leads to greater sensitivity according to personal risk factors and does not create undue alarm amongst those meeting health recommendations (Griffin & Harris, 2011). Our findings show, for the first time, that this sensitization extends to attempts to persuade relatively low-risk individuals that they nevertheless need to improve in order to meet challenging guidelines. That is, rather than necessarily increasing acceptance whenever messages claim individuals are at risk, self-affirmation appears to facilitate sensitivity to how reasonable messages are, given the disparity between achieved and recommended behaviour. There was evidence of the predicted moderating role of physical activity on self-affirmation effects for message acceptance, message derogation, response-efficacy, and self-efficacy, but not for perceived risk of heart disease, behavioural expectations, or self-reported physical activity.

As predicted, self-affirmed individuals who were already relatively active were more sceptical about whether it was necessary or feasible for them to get at least an hour of MVPA on every day of the week. These participants were less accepting of the message, more inclined to derogate it, and reported lower self- and response-efficacy for meeting the recommendations. This suggests that in certain circumstances, self-affirmation may lead to more realistic appraisals of challenging targets. This realism may ultimately prove beneficial, reducing the risk of demoralization and dissatisfaction with outcomes that can lead to failure in the maintenance of behaviour (Rothman, 2000).

It is interesting to note that the threshold at which self-affirmation began to be associated with decreased message acceptance was close to the average physical activity level of the participants. Comparative risk judgements for heart disease have been shown to be relatively accurate (Radcliffe & Klein, 2002), and it is possible that – as well as the target-performance disparity – comparative standing on the behaviour influenced whether or not calls for behaviour change were judged as reasonable. Indeed, research has shown that perceived comparative risk of health threats predicts attitudes towards taking protective action after controlling for perceptions of absolute risk (Klein, 2002). Peer comparison information has also been shown to be more important than recommended levels of the behaviour when both types of information are provided (Schmiege, Klein, & Bryan, 2010).

Considering also that participants who were more than averagely active were reaching the more well-known recommendation for adults (2.5 hr of moderate exercise per week), it is easy to see why greater open-mindedness might lead young people to be sceptical of the advice that it is necessary to exercise for 60 min/day. In previous research, self-affirmation has been linked to reliance on existing beliefs and greater self-confidence (Briñol, Petty, Gallardo, & DeMarree, 2007). Thus, in the current study, it possible that self-affirmed participants felt more able to decide whether recommendations were reasonable, with active participants becoming more doubtful about whether behaviour change was easy or necessary. Self-confidence might have also encouraged greater leniency when active participants were judging small disparities between their performance and the activity guidelines presented (McFarland & Ross, 1982; Schneider, 2001). Although we did not compare to a neutral message control, such effects suggest that caution may be needed in applying self-affirmation with lower-risk individuals and challenging health recommendations.

Amongst those whose activity levels were more discrepant with recommendations, self-affirmation appeared to go some way towards increasing persuasion. As predicted, self-affirmation was associated with significantly higher self-efficacy, and there were trends for greater message acceptance and less derogation compared with the control condition. This is in line with our prediction that relatively inactive individuals will focus on being *more* physically active, whereas those closer to achieving the recommendations might be more focused on the specific target. The absence of stronger effects may reflect the fact that the specific target was restated in the questions (e.g., ‘Getting at least 60 min of moderate-vigorous physical activity a day is effective in reducing the risk of heart disease’). That is, whilst encouraging open-mindedness amongst those whose activity is considerably below the recommended level may facilitate acceptance of the ease of *increasing* physical activity, it is possible that reminders of the target minimized this effect.

Another issue deserving of consideration is whether rejection of challenging messages induced by self-affirmation has behavioural implications. We found the expected moderation effect for self-efficacy – typically a good predictor of behaviour (Sheppard, Hartwick, & Warshaw, 1988; Williams & French, 2011) – but no effects on self-reported behaviour 1 week later. Whilst some studies have reported benefits of self-affirmation for behaviour change (e.g., Epton & Harris, 2008; Jessop, Simmonds, & Sparks, 2009; Pietersma & Dijkstra, 2011), others have failed to find effects on self-reported behaviours 1 week later (Harris & Napper, 2005; Harris, Mayle, Mabbott, & Napper, 2007; Reed & Aspinwall, 1998). In their review, Harris and Epton (2009) note that this is not a problem unique to the self-affirmation literature, and indeed, that it may be best to consider self-affirmation to be a motivational manipulation, which requires bolstering with procedures known to target volitional or goal-striving processes.

### Limitations

In common with other self-affirmation studies, we relied on self-reported behaviour. Whilst the items in the PACE+ Adolescent Physical Activity measure used here have been shown to have good convergent reliability with objective modes of assessment **(**Prochaska *et al*., 2001), it remains possible that T2 reports in both conditions were exaggerated following exposure to the physical activity recommendations. This may have undermined the ability of the study to detect differences between conditions. Future studies should aim to supplement self-reports with data from accelerometers or other measures less prone to error.

A further limitation is that the health message in this study highlighted the risk of heart disease, and although attempts were made to explain the personal relevance of this issue (e.g., ‘fatty build-up in the coronary arteries occurs throughout life’), clinical symptoms do not usually appear until later life (Janz *et al*., 2005; Riddoch *et al*., 2004; Twisk, 2001) and adolescents can find it hard to relate to diseases that characterize the adult population (Wistoft, 2010). This may explain the absence of self-affirmation effects on perceived susceptibility. It would be worthwhile to explore moderation of self-affirmation effects in the context of threats that are likely to be central to the identity of adolescents, such as weight-gain or other appearance-related consequences. More generally, there is a need for further examination of this understudied population within self-affirmation research (McQueen & Klein, 2006).

### Conclusions

In this study, self-affirmation was associated with lower levels of acceptance when a message informed relatively active adolescents that (1) they were at increased risk of heart disease if they did not engage in at least an hour of MVPA every day and (2) it would be easy to achieve this target. There was also evidence that self-affirmation increased message acceptance amongst less active participants, although findings were less clear in this group. We interpret these results as extending previous findings that self-affirmation ‘calibrates’ or sensitizes responses to health messages according to message quality and personal relevance (e.g., Correll *et al*., 2004; Griffin & Harris, 2011). It is likely that self-affirmation increased open-mindedness through its effects on self-integrity, but there may be alternative explanations. As Harris and Epton (2009) note, however, ‘we currently know more about what does not appear to mediate the effects (e.g., explicit positive mood, boosts to state self-esteem, agreeableness) than what does’ (p. 973).

Self-affirmation can be a valuable tool for encouraging uptake of health messages – but only when open-minded appraisals of their content can reasonably be expected to lead to acceptance. When recommendations are so challenging that they are likely to be considered unrealistic even by those who are not in a defensive mindset, self-affirmation may encourage message rejection.

Although we cannot say whether the message used had a detrimental effect (because there was no neutral message condition), our findings provide some indication that caution is needed when deciding whether and how to disseminate challenging health guidelines. Particularly when the majority of the target population fails to come close to the recommended behaviour, it might be advantageous to recognize dose–response relations such as those found for physical activity and health (Janssen & LeBlanc, 2010). People prefer goals that achieve desirable outcomes, but that they also perceive to be feasible (e.g., Ajzen, 1991; Bandura, 1997), and having targets that are out of reach can undermine participation in physical activity (Brawley & Latimer, 2007).

## Footnotes

1 Interpretation of effect sizes: Cramér's *V*: .01 = small effect size, .06 = medium, .16 = large. Eta squared: .01 = small, .06 = medium, .14 = large (Stevens, 2002).

2 The moderator (physical activity) was not mean centred because it has repeatedly been shown to be unnecessary to mean centre or standardize lower-order variables prior to construction of products used in testing interactions (e.g., Echambadi & Hess, 2007; Irwin & McClelland, 2001; Kromrey & Foster-Johnson, 1998; Whisman & McClelland, 2005). In a comparable analysis in which the moderator was mean centred, the results were identical.
